# Multidisciplinary Treatment for Cecal Cancer and Metachronous Liver Metastases in a Patient with Primary Autoimmune Neutropenia

**DOI:** 10.70352/scrj.cr.25-0527

**Published:** 2025-10-29

**Authors:** Kyoichi Okawa, Hiroyuki Yoshidome, Emi Togasaki, Satoshi Ambiru

**Affiliations:** 1 Department of Surgery, Oami Municipal Hospital, Oami-Shirasato, Chiba, Japan; 2 Department of Hematology, Oami Municipal Hospital, Oami-Shirasato, Chiba, Japan

**Keywords:** autoimmune neutropenia, colorectal cancer, hepatectomy, chemotherapy, infection, liver regeneration

## Abstract

**INTRODUCTION:**

Autoimmune neutropenia (AIN) is a rare disease that causes neutropenia due to autoantibodies directed against neutrophils. Neutropenia is associated with an increased risk of infection, such as surgical site infection or febrile neutropenia. To our knowledge, however, there are few reports of surgical or neoadjuvant chemotherapeutic treatments for colorectal cancer with AIN. Herein, we present a case with AIN undergoing multidisciplinary treatment of cecal cancer and metachronous liver metastases.

**CASE PRESENTATION:**

A 74-year-old woman with AIN presented to our hospital with epigastric pain lasting for 3 weeks. Abdominal CT showed obstructive cecal cancer and swollen regional lymph nodes. She had been under observation for primary AIN in the hematology department in our institution. The blood test revealed white blood cell count of 2300/μL, neutrophil count of 19.4%, and thus absolute neutrophil count of 446/μL. Granulocyte-colony stimulating factor (G-CSF; filgrastim 75 μg) was administered to lower the risk of infectious complications before surgery. After the absolute neutrophil count levels increased sufficiently, laparoscopic ileocecal resection was performed. Pathological findings showed T3N1aM0, pStage IIIB (UICC 8th edition), HER2 score 0, and a RAS codon 12S mutation. Six months after curative surgery, multiple liver metastases appeared. A total of 8 cycles of mFOLFOX6 with bevacizumab, combined with G-CSF (filgrastim 75 μg) were administered. After preoperative chemotherapy, the patient underwent right anterior sectionectomy and partial resection of segment 6. She was uneventful in the postoperative course. Throughout the perioperative period and chemotherapy, no infectious complications were observed.

**CONCLUSIONS:**

The administration of G-CSF to prevent neutropenia allowed the patient with AIN to safely undergo multidisciplinary treatment.

## Abbreviations

AIN
: autoimmune neutropenia
ANC
: absolute neutrophil count
CEA
: carcinoembryonic antigen
FN
: febrile neutropenia
G-CSF
: granulocyte-colony stimulating factor
HNA
: human neutrophil antigens
SLE
: systemic lupus erythematosus

## INTRODUCTION

Neutropenia is defined as a condition demonstrating an ANC that decreases to less than 1500/μL.^1)^ In general, neutropenic patients are at risk of severe infections, such as surgical site infection or FN, which is especially life-threatening. Surgical oncologists usually postpone surgery or chemotherapy administration for such patients. AIN is defined by a reduction of ANC caused by autoantibodies against HNA.^2–8)^ The HNA-1 antigen, expressed by the neutrophil-specific Fcγ IIIb receptor (CD16), is most commonly targeted, mainly in its epitopes HNA-1a and HNA-1b.^2–6)^ AIN can be divided into primary and secondary forms. The causes of secondary AIN are associated with autoimmune disorders such as SLE or Felty’s syndrome, infectious diseases, malignancy, or medications.^4)^ The management of secondary AIN in adults requires extensive treatment, including the use of G-CSF and prophylactic antibiotics, whereas the management of primary AIN is usually supportive, with antibiotics used only for infectious episodes, as the bone marrow’s response to infection is typically preserved.^2,5,8)^ However, there are few reports of abdominal surgery in a patient with primary AIN and the perioperative infection risk remains unclear. We report a case of multidisciplinary treatment for a cecal cancer patient with primary AIN.

## CASE PRESENTATION

**Fig. 1:**
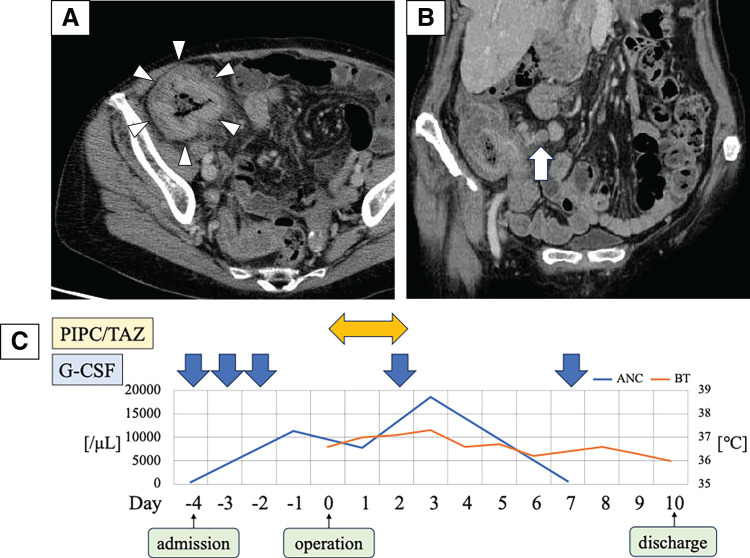
(**A**, **B**) Wall thickness of the cecum (arrow heads) and swollen lymph nodes (arrow). (**C**) Perioperative course of laparoscopic ileocecal resection. ANC and BT were demonstrated during perioperative course. G-CSF (filgrastim 75 μg) was administered at 2, 3, and 4 days before surgery, and on the 2nd and 7th PODs. ANC, absolute neutrophil count; BT, body temperature; G-CSF, granulocyte-colony stimulating factor; PIPC/TAZ, piperacillin/tazobactum

A 74-year-old female presented to our hospital with epigastric pain lasting for 3 weeks. Abdominal examination revealed a palpable mass in the right lower abdomen. The blood test revealed white blood cell count of 2300/μL, neutrophil count of 19.4% (ANC 446/μL), hemoglobin at 9.9 g/dL, and CEA 7.1 ng/dL. Abdominal CT showed obstructive cecum cancer and swollen regional lymph nodes (**Fig. 1A**, **1B**).

When she was 70 years old, a routine medical screening revealed neutropenia according to her medical history. She had not been on any medications and did not have any autoimmune disorders or other known factors associated with neutropenia. The bone marrow test showed neutrophile precursor cells, and she responded positively to treatment with G-CSF. The presence of HNA-1a antibodies was confirmed, leading to a diagnosis of primary AIN. Since then, she has been under observation for primary AIN in the hematology department in our institution.

**Fig. 2:**
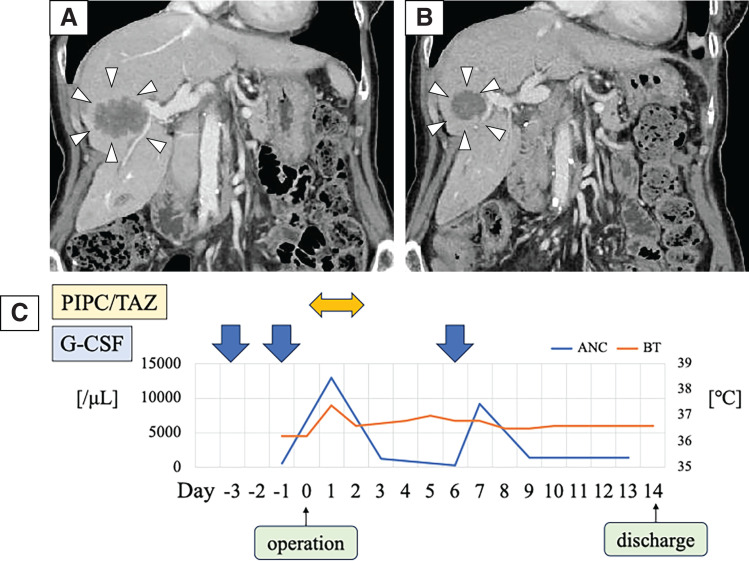
(**A**) Six months after ileocecal resection. 46 × 44 × 30 mm liver metastasis invading the right anterior Glisson’s capsule. (**B**) After 8 cycles of mFOLFOX6 with bevacizumab, the tumor size reduced to 30 × 31 × 26 mm. (**C**) Perioperative course of hepatectomy. ANC and BT were demonstrated during perioperative course. G-CSF (filgrastim 75 μg) was administered at 1 and 3 days before surgery, and on the 6th POD. ANC, absolute neutrophil count; BT, body temperature; G-CSF, granulocyte-colony stimulating factor; PIPC/TAZ, piperacillin/tazobactum

**Fig. 3:**
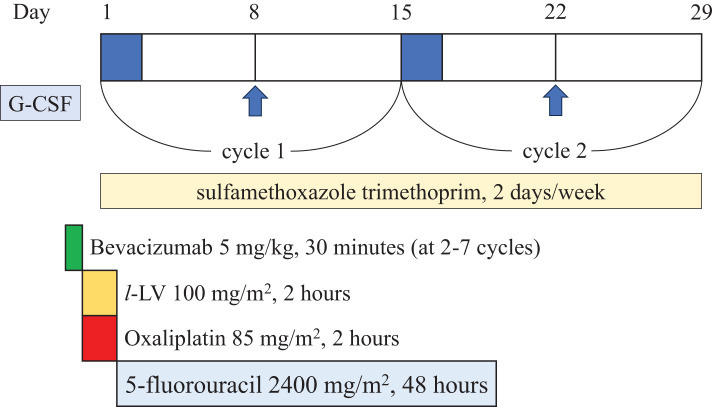
Time course of mFOLFOX6 with bevacizumab, combined with G-CSF (oxaliplatin 85 mg/m^2^, but without 5-fluorouracil bolus injection). G-CSF (filgrastim 75 μg) was administered at day 8. Sulfamethoxazole trimethoprim was continuously taken twice/week. G-CSF, granulocyte-colony stimulating factor

Due to the obstruction, it was considered that urgent surgery was needed. Due to concerns about perioperative infectious complications, G-CSF (filgrastim 75 μg) was administered for 3 consecutive days before surgery (2, 3, and 4 days before surgery), and piperacillin/tazobactam was chosen as a broad-spectrum antibiotic (**Fig. 1C**). Once the ANC level had sufficiently increased, laparoscopic ileocecal resection was performed (**Fig. 1C**). The operative time was 188 minutes. On the 2nd and 7th PODs, G-CSF (filgrastim 75 μg) was administered. She discharged on the 10th POD without any complications. Pathological findings showed T3N1aM0, pStage IIIB (UICC 8th edition), HER2 score 0, and a RAS codon 12S mutation. Postoperative adjuvant chemotherapy was not administered because the patient did not consent. Six months after surgery, the abdominal CT scan revealed multiple liver metastases. The largest tumor was 46 × 44 × 30 mm, suspected to invade the right hepatic vein and the right anterior Glisson’s capsule (**Fig. 2A**). Since liver metastases occurred only 6 months after initial resection for primary tumor, upfront liver resection was unfavorable from the standpoint of oncological aspect for curative surgery. Therefore, conversion chemotherapy consisting of 8 cycles of mFOLFOX6 (oxaliplatin 85 mg/m^2^, but without 5-fluorouracil bolus injection) with bevacizumab, combined with G-CSF (filgrastim 75 μg) was administered at day 8 (**Fig. 3**). Chemotherapy was performed every 2 weeks without delay. Sulfamethoxazole trimethoprim was continuously taken twice/week as hematologist indicated from the initial diagnosis of AIN (**Fig. 3**). After chemotherapy, the largest tumor size reduced to 30 × 31 × 26 mm (**Fig. 2B**). CT volumetry indicated that a future remnant liver volume of 700 mL (66.9% of the total liver volume) was sufficient for liver surgery, allowing right anterior sectionectomy and partial resection of segment 6. As with the initial procedure, surgery was performed following an increase in ANC following G-CSF (filgrastim 75 μg) administration at 1 and 3 days before surgery (**Fig. 2C**). The operative time was 459 minutes. Blood loss was 845 mL. On the 6th POD, G-CSF (filgrastim 75 μg) was administered. She was discharged on the 14th POD without any complications. She was alive with liver recurrence for 2 years and 7 months from the initial resection.

## DISCUSSION

Primary AIN is a rare disease for both children and adults. In newborns with primary AIN, historical data suggest an annual prevalence at 1/100000,^6)^ while data from the Italian neutropenia registry showed the frequency of primary AIN in premature babies to be 13.2%.^4)^ In an adult, the prevalence is considered much lower than in newborns.^7)^ It remains unclear due to the scarcity of anti-neutrophil antibody screening in the diagnostic work-up of most adult neutropenia patients, and the infrequent reporting of adult cases in registries.^3)^ For example, Shastri and Logue^9)^ reported that anti-neutrophil antibodies were detected in 36% of the sera from 121 adult patients with chronic idiopathic neutropenia. Similary, Sicre de Fontbrune et al.^10)^ demonstrated that the neutrophil autoantibodies were detected in 34.8% of 108 adults with severe chronic primary neutropenia. In this case, there was no evidence of secondary AIN, and HNA-1a antibody was examined by the Department of Pediatrics of Hiroshima University.

Abdominal surgery for neutropenic patients is always challenging. A previous report showed that postoperative mortality rate was 41%.^11)^ However, some studies have suggested the possibility of safe treatment in such cases by administration of G-CSF. Emergency surgery in neutropenic patients carries a substantial risk of severe complications. In contrast, elective surgery following an increase in ANC levels can provide outcomes comparable in safety to those of non-neutropenic patients.^12–14)^ In a recent report, the 30-day mortality rate of surgery in neutropenic patients was reported to be 11.8%.^13)^ We administered G-CSF both before and after surgery to sufficiently raise the ANC levels during the 5th to 7th PODs, a period of high risk for anastomotic failure. This approach was intended to prevent a potentially fatal outcome. Adachi et al.^15)^ demonstrated a case of AIN undergoing breast cancer surgery. In this case, G-CSF administration was unable to raise ANC, and the patient had fever (38.6°C) after surgery. In our case, she responded well to G-CSF administration with sufficient ANC levels, and she did not have high grade fever after surgery.

In chemotherapy as well, neutropenia significantly increases the risk of infectious complications, such as FN, and certain chemotherapy regimens are known to induce this condition. For patients with myeloma and lymphoma, the G-CSF response is not only associated with infectious risk following high-dose chemotherapy, but also serves as a superior predictive factor compared with the duration of neutropenia.^16)^ In patients with primary AIN, the G-CSF response is typically preserved because their bone marrow is normocellular or hypercellular. Trus et al.^17)^ reported a case of adjuvant chemotherapy for triple-negative breast cancer in a patient with primary AIN. They administered FEC-100 (5-fluorouracil, epirubicin, cyclophosphamide) at a 20% reduced dose, combined with G-CSF and cyclosporin A. Although she developed FN twice during the period, no focus of infection was identified. In this case, G-CSF was administered on the 8th day of each cycle of mFOLFOX6 with bevacizumab because neutropenia typically occurs between the 7th and 10th days of the regimen. Since 5-fluorouracil bolus injection was known to be related to reduction in neutrophil counts, we did not use 5-fluorouracil bolus injection. However, we did not reduce the dose of oxaliplatin. As a result, FN did not occur during chemotherapy.

Thus, we successfully treated a cecal cancer patient with primary AIN. However, the optimal timing for G-CSF administration remains uncertain because few reports exist on surgery or chemotherapy for patients with primary AIN; this presents a limitation of this report. It is necessary to discuss the most appropriate timing for G-CSF administration with hematology specialists.

## CONCLUSIONS

In patients with primary AIN, G-CSF administration may help reduce the risk of infectious complications following surgery or chemotherapy. This is the first case report on multidisciplinary treatment for a primary AIN patient with colorectal cancer, to our knowledge.

## DECLARATIONS

### Availability of data and materials

Data will be made available on reasonable request.
